# Comprehensive Pan-Cancer Analysis of IRAK Family Genes Identifies IRAK1 as a Novel Oncogene in Low-Grade Glioma

**DOI:** 10.1155/2022/6497241

**Published:** 2022-02-15

**Authors:** Jing Li, Yuchen Sun, Yuan Ma, Xu Zhao, Xuanzi Sun, Yuzhu Wang, Xiaozhi Zhang

**Affiliations:** The First Affiliated Hospital of Xi'an Jiaotong University, Department of Radiation Oncology, Xi'an 710061, China

## Abstract

**Background:**

The interleukin-1 receptor-associated kinases (IRAK) family genes, indispensable mediators of interleukin-1 receptor (IL1R) and Toll-like receptor (TLR)-inflammatory signaling, may be involved in the biological function of human cancers due to the crucial roles of inflammation in tumor development. Though a little research has demonstrated the function of individual IRAK family members in specific tumors, comprehensive analysis is still lacking in pan-cancer.

**Methods:**

We analyzed the mRNA expression landscape, mutation, and prognosis value of IRAK genes based on The Cancer Genome Atlas (TCGA), cBioPortal, GlioVis, and Rembrandt databases. The correlation between the expression of IRAK genes and tumor microenvironment (TME), Stemness score, and immune subtypes was explored. Western blot, cell proliferation, apoptosis, migration assays, and xenograft models were utilized in this study.

**Results:**

We found that the expression of IRAK genes extensively changed and was related to patient survival in pan-cancer. Besides, IRAK family genes were correlated with TME, Stemness score, and immune subtypes in most cases. Given that high expression of all IRAK family members predicted poor prognosis in low-grade glioma (LGG), the oncogenic function of the highest expressed IRAK1 in LGG has been confirmed in vitro and in vivo. IRAK1 was uncovered to inhibit cell apoptosis and augment malignancy of LGG in vitro and in vivo.

**Conclusion:**

These findings revealed the potential targets of IRAK family genes in pan-cancer and provided insights for further investigation of IRAK1 as a novel oncogenic gene in LGG.

## 1. Introduction

Interleukin-1 receptor-associated kinases (IRAK) play a crucial role in cellular apoptosis, inflammation, and differentiation by regulating Toll-like receptor (TLR) and interleukin-1 receptor (IL1R) signaling [1]. The IRAK family is comprised of IRAK1, IRAK2, and IRAK4, which are ubiquitously expressed in various human cell types, and IRAK3 (also named IRAK-M), induced only in monocytes and macrophages [2, 3]. Except for IRAK4, all IRAK members share a similar C-terminal domain, which is required for the activation of TNF receptor-associated factor 6 (TRAF6) and downstream NF-*κ*B, p38, and JNK MAPKs signalings [4, 5].

Since the contradictory roles of inflammation in tumorigenesis and progression, whether IRAK family genes exhibit tumor-supportive or tumor-suppressive responses remains relatively unknown [6]. IRAK1, the first member to be identified among the IRAK family, localizes to both the cytoplasm and nucleus of cells. IRAK1 was reported to be overexpressed and correlated with advanced tumor stages and poor patient prognosis in hepatocellular carcinoma (HCC) [7], lung cancer [8], and endometrial carcinoma [9]. Also, IRAK1 could augment the self-renewal, tumorigenicity, and chemoresistance of tumor-initiating cells in HCC [7, 10]. Alternatively, deregulated expression of IRAK2 potentially acts as a tumor suppressor to counterbalance oncogenic Smurf1 by dictating endoplasmic reticulum in colon cancer [11]. IRAK-M, the only member of the IRAK family who lacks kinase activity, prevents the formation of the IRAK1/TRAF6 complex and hence inhibits the activation of downstream NF-*κ*B signaling [12]. IRAK-M was revealed to facilitate cancer progression by regulating macrophage activity and creating a more favorable tumor microenvironment (TME) [13, 14]. Constitutive IRAK4 activation mediates tumorigenesis and chemoresistance in pancreatic ductal adenocarcinoma [15] and colorectal cancer [16]. An integrative study of IRAK family genes may unveil novel prognosis and therapeutic targets for human cancers.

In this study, we analyzed the expression level and prognosis value of the IRAK family in pan-cancer. The potential association between IRAK family genes and TME, stemness, and immune subtypes was evaluated. The findings of this study highlight the crucial role of the oncogenic gene IRAK1 in low-grade glioma (LGG).

## 2. Materials and Methods

### 2.1. Data Collection and cBioPortal

The RNA-Seq (RNA SeqV2 RSEM) and corresponding clinical data were obtained from the Xena browser (version 07-20-2019, https://xenabrowser.net/datapages/). The data for 33 types of cancer were downloaded, including ACC, BLCA, BRCA, CESC, CHOL, COAD, DLBC, ESCA, GBM, HNSC, KICH, KIRC, KIRP, LAML, LGG, LIHC, LUAD, LUSC, MESO, OV, PAAD, PCPG, PRAD, READ, SARC, SKCM, STAD, TGCT, THCA, THYM, UCEC, UCS, and UVM. The differential expression and survival curves were investigated for 18 cancer types with more than 5 normal adjacent tissues. The clinical-stage information of LGG patients in the TCGA cohort was downloaded from GlioVis (version 01-15-2020, https://gliovis.bioinfo.cnio.es/). The expression levels of IRAK family genes in non-tumor and tumor tissues from the Rembrandt database were obtained from the Chinese Glioma Genome Atlas (CGGA, version 06-14-2020, https://www.cgga.org.cn/). Genetic mutations of the IRAK family and their association with overall survival (OS) and progression-free survival (PFS) of LGG patients were analyzed by the cBioPortal online tool (version 3.7.19, https://www.cbioportal.org/).

### 2.2. Tumor Microenvironment Analysis and Stemness

The Immune score, Stromal score, and Estimate score were calculated by the ESTIMATE algorithm with the R-package “estimate” and “limma” in pan-cancer. DNA stemness score (DNAss) was downloaded from the Xena browser (version 07-20-2019, https://xenabrowser.net/datapages/). The association analyses between IRAK gene expression and Immune score, Stromal score, Estimate score, and DNAss were assessed by using Spearman correlation test and R-package “corrplot”.

### 2.3. Cell Lines and Cell Culture

The human Hs683 and SW1088 low-grade glioma cell lines employed in the study were purchased from the Cell Bank of Type Culture Collection of the Chinese Academy of Science (Shanghai, China). Cells were cultured in Dulbecco's modified Eagle's medium (DMEM; HyClone, Logan, USA) mixed with 10% fetal bovine serum (FBS; Gibco, NY, USA) and 1% penicillin-streptomycin (HyClone, Logan, USA) at 37°C incubators with 5% CO_2_. To knock down IRAK1 expression, the short hairpin (shRNA) was designed by GeneChem Co., Ltd (Shanghai, China). The target sequences of IRAK1 shRNA and the negative control (NC) were 5′-GCCACCGCAGATTATCATCAA-3′ and 5′-TTCTCCGAACGTGTCACGT-3′, respectively. The transfection was performed according to the manufacturer's instructions.

### 2.4. Cell Proliferation Assay

Cell proliferation was evaluated by Cell Counting Kit-8 assays (CCK-8, TargetMol). Briefly, 3,000 glioma cells in 100 *μ*l medium were seeded into 96-well plates and cultured for the indicated time points. Next, 10 *μ*l of CCK-8 solution was added to each well, and cells were incubated for another 4 h. Finally, the optical density (OD) value of each sample was measured at 450 nm. Five replicate wells were conducted in each group.

### 2.5. Cell Apoptosis Assay

The apoptosis rate of glioma cells was tested by Annexin V-APC/7-AAD detection kit (BD Bioscience, USA). Glioma cells were collected and washed with cold PBS, resuspended in 100 *μ*l 1× binding buffer, and incubated at room temperature for 15 min away from light. Then, cells were stained with 5 *μ*l Annexin V-APC and 5 *μ*l 7-AAD, supplemented with 400 *μ*l 1× binding buffer. The samples were immediately subjected to a flow cytometer to test the apoptosis rate.

### 2.6. Transwell Migration Assay

For the cell migration assay, 5 × 10^4^ cells suspended in a 200 *μ*l serum-free medium were added to the upper chambers of 24-well transwell chambers (Corning, MA, USA). Subsequently, 600 *μ*l DMEM supplemented with 20% FBS was added to the bottom of the plates. After the incubation for 24 h at 37°C, the membrane was fixed with methanol and stained with 0.5% crystal violet for 15 min. Cells were counted in three independent photographed fields.

### 2.7. Western Blot Analysis

Total protein of cells was harvested in RIPA lysis buffer (Sigma-Aldrich, MO, USA) containing a protease inhibitor. The protein concentration of the whole-cell lysate was determined by the BCA Protein Assay (Sigma-Aldrich, MO, USA). Then, protein samples were separated by standard 10% SDS-PAGE gel electrophoresis and transferred to polyvinylidene difluoride (PVDF) membranes (Millipore, MA, USA). The membranes were then blocked in 5% fat-free milk for 1 h at room temperature, dissolved with Tris-buffered saline-Tween (TBST). Then, all membranes were incubated overnight at 4°C with the primary antibodies: anti-IRAK1 (#4504; Cell Signaling Technology, MA, USA; 1 : 1000), anti-E-cadherin (20874-1-AP, Proteintech, 1 : 5000), anti-N-cadherin (22018-1-AP, Proteintech, 1 : 2000), anti-Vimentin (10366-1-AP, Proteintech, 1 : 1000), and anti-GAPDH (10494-1-AP, Proteintech, 1 : 5000). After washing with TBST three times, the membranes were then incubated with the secondary antibody for 1.5 h at room temperature. The bands were finally visualized using an ECL reagent (Millipore, MA, USA) after washing four times with TBST solution.

### 2.8. Xenograft Tumor Model

The animal experiment in this study was approved by the Institutional Animal Care and Use Committee of the First Affiliated Hospital of Xi'an Jiaotong University. Tumor cells (8 × 10^6^ cells/100 *μ*l) were subcutaneously injected into the right flanks of 4-week-old female BALB/c nude mice. The length (a) and width (b) of the xenograft tumors were assessed every 4 days. The tumor volume (V) was calculated by the following formula: V = ab^2^/2. After 20 days, the mice were sacrificed, and the xenograft tumors were isolated and measured.

### 2.9. Statistical Analysis

The differential expression of IRAK family genes between tumor and adjacent normal tissue was analyzed by the Wilcoxon test. Kaplan–Meier analysis with log-rank tests was utilized to perform survival curves. The hazard ratio for the correlation analysis for IRAK gene expression and survival was calculated by Cox regression. The comparison of IRAK family genes among various immune subtypes was demonstrated by the Kruskal–Wallis test. All analyses were performed using R 4.0.5 (https://cran.r-project.org/doc/FAQ/R-FAQ.html#Citing-R). *P* < 0.05 was considered statistically significant.

## 3. Results

### 3.1. Expression Levels of IRAK Family in Pan-Cancer

**Figure 1:**
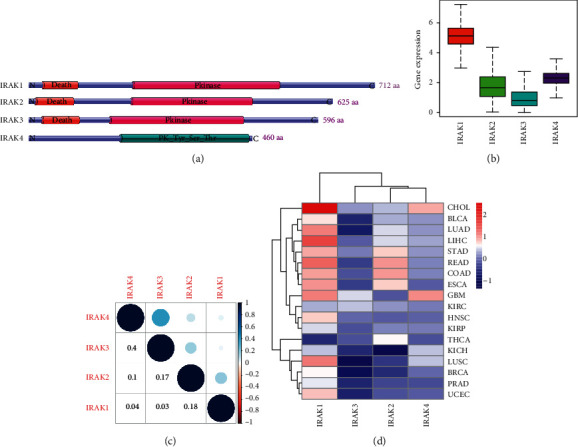
IRAK family gene expression levels in pan-cancer from TCGA. (a) The protein domain structure of IRAK family members and the pattern diagram were constructed using IBS 1.0 (http://ibs.biocuckoo.org/index.php). (b) Boxplot to show the expression of IRAK genes across all 33 cancer types. (c) The correlation among the four IRAK family members based on Pearson's correlation test in pan-cancer. (d) Heatmap showing differential IRAK gene expression across 18 cancer types, which have more than 5 normal tissues to compare. The red and blue colors represent the high and low expression, respectively.

The IRAK family genes had definite genomic chromosomal locations shown in Table 1. The protein kinases of IRAK1, IRAK2, and IRAK3 are described to share similar domains, including a Pkinase domain and an N-terminal death domain, which is vital for dimerization and MyD88 interaction. IRAK4 contains a PK_Tyr_Ser_Thr domain (Figure 1(a)).

**Figure 2:**
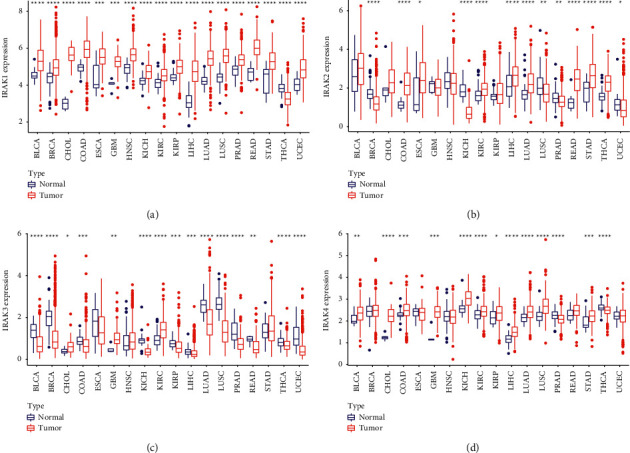
The differential expression levels of the IRAK family genes between tumor and adjacent normal tissue in 18 cancer types. (a) IRAK1, (b) IRAK2, (c) IRAK3, and (d) IRAK4. The red and blue rectangle boxes indicate gene expression levels in tumor and normal tissue, respectively.  ^*∗*^*P* < 0.05,  ^*∗∗*^*P* < 0.01,  ^*∗∗∗*^*P* < 0.001,  ^*∗∗∗∗*^*P* < 0.0001. BLCA, bladder urothelial carcinoma; BRCA, breast invasive carcinoma; CHOL, cholangiocarcinoma; COAD, colon adenocarcinoma; ESCA, esophageal carcinoma; GBM, glioblastoma multiforme; HNSC, head, and neck squamous cell carcinoma; KICH, kidney chromophobe; KIRC, kidney renal clear cell carcinoma; KIRP, kidney renal papillary cell carcinoma; LIHC, liver hepatocellular carcinoma; LUAD, lung adenocarcinoma; LUSC, lung squamous cell carcinoma; PRAD, prostate adenocarcinoma; READ, rectum adenocarcinoma; STAD, stomach adenocarcinoma; THCA, thyroid carcinoma; UCEC, uterine corpus endometrial carcinoma.

To reveal the expression levels of IRAK family genes, we downloaded mRNA expression data of 33 cancer types available from the TCGA database. IRAK1 showed a higher expression level. IRAK2 and IRAK4 were moderately expressed, and IRAK3 expression was the relatively lowest in pan-cancer (Figure 1(b)). Further, Pearson correlation tests of IRAK family members indicated that they were positively correlated with each other to varying degrees. Among them, the pair of IRAK3 and IRAK4 presented the highest positive correlation (*r* = 0.4, Figure 1(c)). We investigated the expression levels of IRAK family genes in 18 cancer types of primary tumors, which have at least 5 normal samples. IRAK1 expression was upregulated in almost all tumors but thyroid carcinoma (THCA) (Figure 1(d) and Figure 2(a)). IRAK2 was more expressed in rectum adenocarcinoma (READ), colon adenocarcinoma (COAD), esophageal carcinoma (ESCA), stomach adenocarcinoma (STAD), THCA, lung adenocarcinoma (LUAD), liver hepatocellular (LIHC), and kidney renal clear cell carcinoma (KIRC). Meanwhile, a lower expression of IRAK2 was observed in uterine corpus endometrial carcinoma (UCEC), prostate adenocarcinoma (PRAD), lung squamous cell carcinoma (LUSC), breast invasive carcinoma (BRCA), and kidney chromophobe (KICH) (Figure 1(d) and Figure 2(b)). IRAK3 expression was higher in glioblastoma multiforme (GBM), KIRC, and cholangiocarcinoma (CHOL). In contrast, lower IRAK3 expression was found in more cancer types, including LIHC, kidney renal papillary cell carcinoma (KIRP), THCA, COAD, READ, KICH, PRAD, bladder urothelial carcinoma (BLCA), UCEC, BRCA, and LUSC (Figure 1(d) and Figure 2(c)). IRAK4 was higher expressed in GBM, CHOL, KICH, LUSC, LIHC, BLCA, LUAD, STAD, COAD, KIRP, and KIRC. At the same time, lower expression of IRAK4 was found in THCA and PRAD (Figure 1(d) and Figure 2(d)). The complexity of the IRAK gene expression spectrum in different cancer types indicated the need for further study of each IRAK gene member.

### 3.2. Association of IRAK Family Gene Expression with Patient Overall Survival

**Figure 3:**
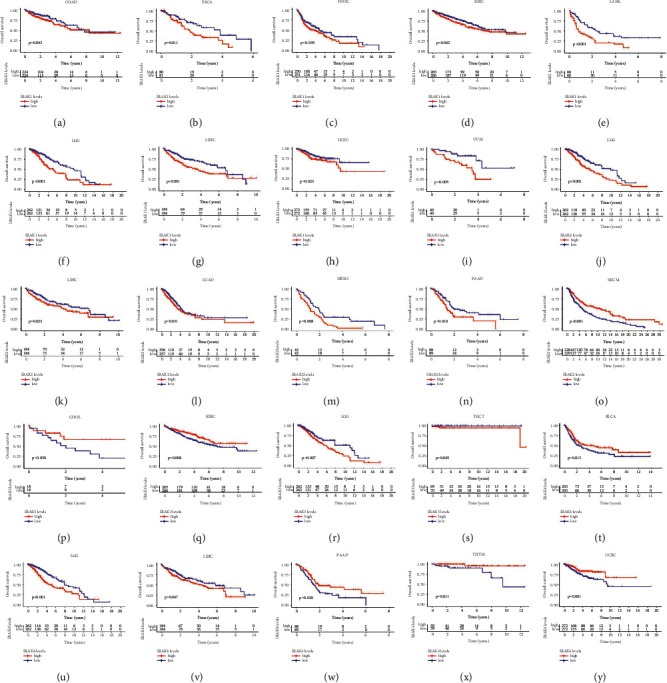
Kaplan–Meier survival curves for IRAK family gene expression significantly correlated with overall survival in pan-cancer. Patients were divided into high (red) and low (blue) expression groups by median as the cutoff value. OS curves for IRAK1 in cancer types: (a) COAD (*n* = 448); (b) ESCA (*n* = 161); (c) HNSC (*n* = 501); (d) KIRC (*n* = 531); (e) LAML (*n* = 132); (f) LGG (*n* = 524); (g) LIHC (*n* = 368); (h) UCEC (*n* = 544); (i) UVM (*n* = 80). OS curves for IRAK2 in cancer types: (j) LGG (*n* = 524); (k) LIHC (*n* = 368); (l) LUAD (*n* = 513); (m) MESO (*n* = 84); (n) PAAD (*n* = 177); (o) SKCM (*n* = 457). OS curves for IRAK3 in cancer types: (p) CHOL (*n* = 36); (q) KIRC (*n* = 531); (r) LGG (*n* = 524); (s) TGCT (*n* = 139). OS curves for IRAK4 in cancer types: (t) BLCA (*n* = 406); (u) LGG (*n* = 524); (v) LIHC (*n* = 368); (w) PAAD (*n* = 177); (x) THYM (*n* = 118); (y) UCEC (*n* = 544).

To identify the prognosis value of the IRAK family genes, we conducted Kaplan–Meier survival curves in pan-cancer from the TCGA database. Herein, the results indicated that the expression of IRAK genes was associated with the survival rate in several cancers (Figure 3). Specifically, patients with higher IRAK1 expression had worse overall survival (OS) than those with lower ones in multiple cancers, including COAD (*n* = 448, *p* = 0.043, Figure 3(a)), ESCA (*n* = 161, *p* = 0.011, Figure 3(b)), HNSC (*n* = 501, *p* = 0.039, Figure 3(c)), KIRC (*n* = 531, *p* = 0.042, Figure 3(d)), LAML (*n* = 132, *p* < 0.001, Figure 3(e)), LGG (*n* = 524, *p* < 0.001, Figure 3(f)), LIHC (*n* = 368, *p* < 0.001, Figure 3(g)), UCEC (*n* = 544, *p* = 0.023, Figure 3(h)), and UVM (*n* = 80, *p* = 0.005, Figure 3(i)). Except for SKCM (*n* = 457, *p* < 0.001, Figure 3(o)), IRAK2 played a detrimental role in five different cancer types, which contained LGG (*n* = 524, *p* < 0.001, Figure 3(j)), LIHC (*n* = 368, *p* = 0.023, Figure 3(k)), LUAD (*n* = 513, *p* = 0.031, Figure3(l)), MESO (*n* = 84, *p* = 0.008, Figure 3(m)), and PAAD (*n* = 177, *p* = 0.010, Figure 3(n)). Besides, IRAK3 acted as a protective prognosis gene in CHOL (*n* = 36, *p* = 0.050, Figure 3(p)) and KIRC (*n* = 531, *p* = 0.008, Figure 3(q)). In contrast, IRAK3 was a high-risk gene in LGG (*n* = 524, *p* = 0.007, Figure 3(r)) and TGCT (*n* = 139, *p* = 0.045, Figure 3(s)). Patients with IRAK4 high expression had a survival advantage compared with low group in BLCA (*n* = 406, *p* = 0.013, Figure 3(t)), PAAD (*n* = 177, *p* = 0.020, Figure 3(w)), THYM (*n* = 118, *p* = 0.011, Figure 3(x)), and UCEC (*n* = 544, *p* = 0.001, Figure 3(y)). Contrarily, IRAK4 was a detrimental prognosis factor in LGG (*n* = 524, *p* < 0.001, Figure 3(u)) and LIHC (*n* = 368, *p* = 0.047, Figure 3(v)).

**Figure 4:**
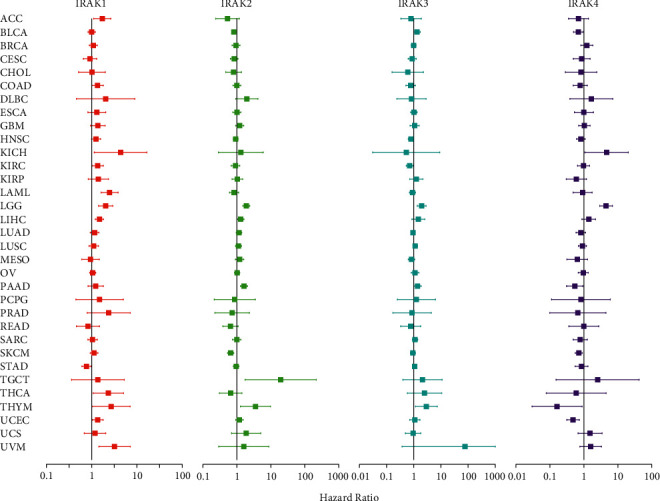
Forest plots for IRAK family gene expression and overall survival in pan-cancer. The hazard ratios (HR) and 95% confidence intervals are shown. HR < 1 and HR > 1 represent a low and high risk, respectively.

Further, Cox analysis in pan-cancer showed that the altered expression of IRAK family genes was correlated with patients' prognosis, which can be varied in different types of cancer (Figure 4 and Table 2). In more detail, IRAK1 predicted shorter OS of patients with ACC, KICH, KIRC, LAML, LGG, LIHC, THCA, THYM, UCEC, and UVM (HR > 1, *P* < 0.05) except for STAD (HR < 1, *P* < 0.05). Increased IRAK2 expression was mainly related to poor prognosis for patients with LGG, LIHC, PAAD, TGCT, and THYM (HR > 1, *P* < 0.05), but predicted survival advantage for BLCA and SKCM (HR < 1, *P* < 0.05). Increased expression of IRAK3 predicted worse survival for patients with BLCA, LGG, PAAD, and THYM (HR > 1, *P* < 0.05). However, IRAK3 predicted a better prognosis for HNSC and KIRC (HR < 1, *P* < 0.05). IRAK4 favored survival for patients with BLCA, PAAD, SKCM, THYM, and UCEC (HR < 1, *P* < 0.05), but was associated with poor survival for LGG and KICH (HR > 1, *P* < 0.05). It is worth noting that high expression of all IRAK family members was associated with poor survival for patients with LGG, which is worth in-depth study.

### 3.3. Correlation of IRAK Gene Expression with Tumor Microenvironment and Stemness

**Figure 5:**
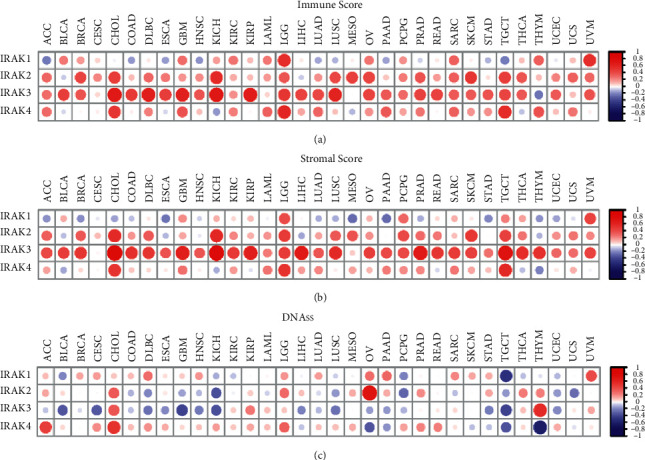
Association of IRAK gene expression with tumor microenvironment and stemness across different cancer types in the TCGA database. Matrix graph for Spearman's correlation test between IRAK family gene expression and Immune score (a), Stromal score (b), and tumor Stemness score (DNAss) (c) in pan-cancer. Red dots indicate a positive correlation, and blue dots indicate a negative correlation.

**Figure 6:**
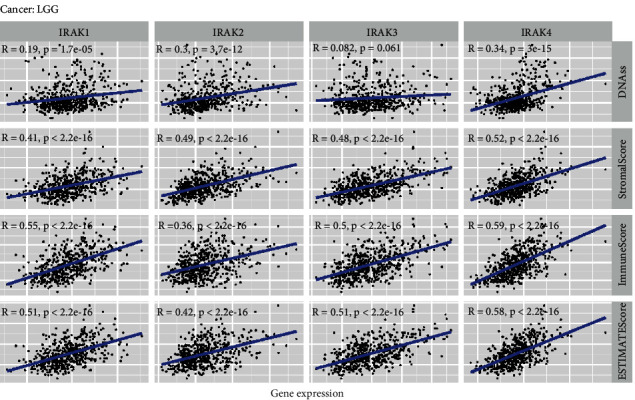
Correlation analysis of IRAK family gene expression with tumor microenvironment and stemness score in LGG. Scatter plots for Spearman correlation tests between IRAK gene expression and DNAss, Immune score, Stromal score, and Estimate score in LGG.

To determine the roles of IRAK family genes in TME, we calculated the Immune score and Stromal score in pan-cancer using the ESTIMATE algorithm. In general, the results showed that IRAK gene expression was mainly positively correlated with Immune score (Figure 5(a)) and Stromal score (Figure 5(b)), reflecting the infiltration levels of immune cells and stromal cells in TME, respectively. Notably, IRAK3 expression had a strong correlation with Immune and Stromal scores in KICH (Immune score, *r* = 0.70; Stromal score, *r* = 0.84) and CHOL (Immune score, *r* = 0.68; Stromal score, *r* = 0.82). Meanwhile, similar result was presented in LGG that IRAK genes were significantly positively associated with immune cell infiltration (IRAK1, *r* = 0.55; IRAK2, *r* = 0.37; IRAK3, *r* = 0.50; IRAK4, *r* = 0.59) and Stromal score (IRAK1, *r* = 0.41; IRAK2, *r* = 0.49; IRAK3, *r* = 0.48; IRAK4, *r* = 0.53, Figure 6). LGG was grouped into astrocytoma, oligodendroglioma, and mixed glioma according to histology-based classification [17]. Similar results were observed in all histology-based subgroups of LGG (Supplementary Figure 1), considering the crucial roles of TME in tumor progression and metastasis, by which IRAK family genes might regulate the malignant behaviors in pan-cancer, especially for LGG.

DNA stemness based on DNA methylation pattern (DNAss) can be applied to measure tumor stemness. IRAK family genes had a significantly varying correlation with DNAss in different cancer types. Specifically, DNAss had a strong positive correlation with IRAK2 in OV (*r* = 0.75) and IRAK3 in THYM (*r* = 0.57). In contrast, IRAK4 was observed to be negatively associated with DNAss in THYM (*r* = −0.61, Figure 5(c)). Interestingly, we found that IRAK genes were all positively correlated with DNAss in LGG, although not significant for IRAK3, suggesting that IRAK family genes tend to facilitate tumor stemness in LGG (Figure 6 and Supplementary Figure 1).

### 3.4. Association between IRAK Gene Expression and Immune Subtypes

**Figure 7:**
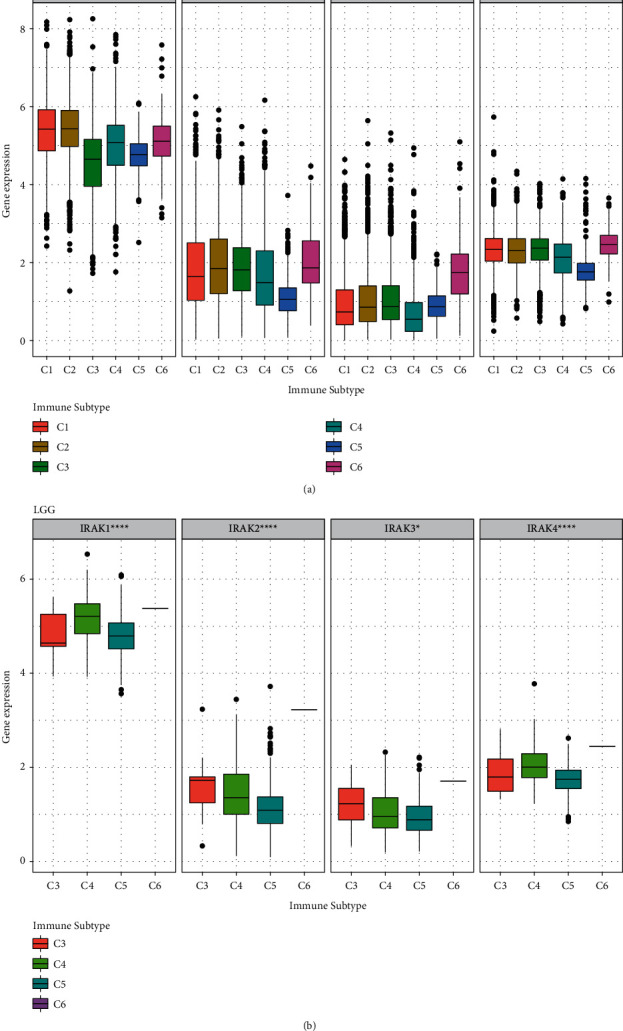
Differential IRAK family gene expression levels across immune infiltrate subtypes in pan-cancer (a) and LGG (b). C1, wound healing; C2, IFN-*γ* dominant; C3, inflammatory; C4, lymphocyte depleted; C5, immunologically quiet; C6, TGF-*β* dominant.  ^*∗*^*P* < 0.05,  ^*∗∗∗∗*^*P* < 0.0001.

Six immune subtypes were identified in solid tumors, including C1 (wound healing), C2 (IFN-*γ* dominant), C3 (inflammatory), C4 (lymphocyte depleted), C5 (immunologically quiet), and C6 (TGF-*β* dominant). Furthermore, patients with C3 and C5 immune subtypes had a better prognosis, while type C4 and C6 patients had distinct survival disadvantages [18]. Correlation analyses were performed to explore the potential association between IRAK family genes and immune subtypes in pan-cancer and LGG, respectively. High expression of IRAK1 was observed in pan-cancer patients with subtypes C1, C2, and C6, implying a tumor-supportive role of IRAK1. Also, IRAK2, IRAK3, and IRAK4 were higher expressed in the C6 subtype in pan-cancer (Figure 7(a)). Likewise, higher expression of IRAK genes were all related to C6 over other infiltrate types in LGG, indicating these genes may play a tumor promoter role in patients with LGG (Figure 7(b)). Particularly, IRAK1 and IRAK4 tend to be significantly higher expressed in C4 and C6 infiltrate types for all histology-based subgroups of LGG patients (Supplementary Figure 2).

### 3.5. Expression and Genetic Mutations of IRAK Family in Low-Grade Glioma

**Figure 8:**
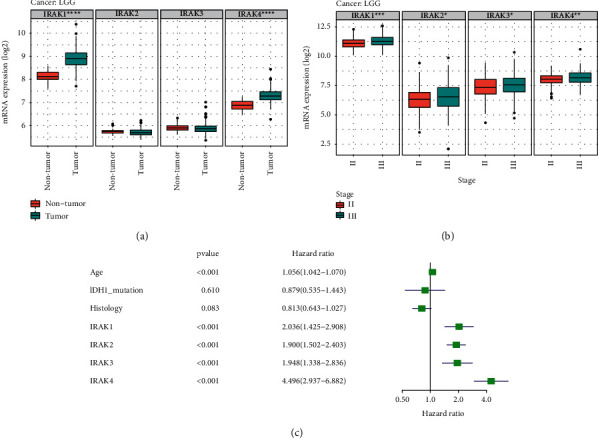
The expression levels of IRAK family genes in LGG. (a) Box plots represent the relative IRAK gene expression in non-tumor (*n* = 28) and tumor tissue of LGG (*n* = 183) in the Rembrandt database. (b) Box plots showing IRAK gene expression levels of stage II (*n* = 226) and stage III (*n* = 244) for LGG patients from the TCGA cohort. (c) A forest map was developed based on univariate Cox regression analysis in the TCGA LGG cohort  ^*∗*^*P* < 0.05,  ^*∗∗*^*P* < 0.01,  ^*∗∗∗*^*P* < 0.001 and  ^*∗∗∗∗*^*P* < 0.0001.

Since the lack of non-tumor samples in the LGG dataset from the TCGA database, the profiles from the Rembrandt database were downloaded to compare the expression abundance of IRAK genes between LGG tumor tissues and normal ones. The results revealed that IRAK1 and IRAK4 expressions in LGG were significantly higher than those in non-tumor tissue (IRAK1, *P* = 1.01 × 10^−14^; IRAK4, *P* = 7.73 × 10^−12^, Figure 8(a)). Moreover, increasing expression levels of IRAK family genes were observed in stage III samples than in stage II samples from the TCGA database (Figure 8(b)). In univariate Cox regression analysis (Figure 8(c)), the IRAK family genes and age could be considered independent prognostic factors in LGG patients from the TCGA cohort. However, histology turned out to be not significantly correlated with OS in LGG patients.

**Figure 9:**
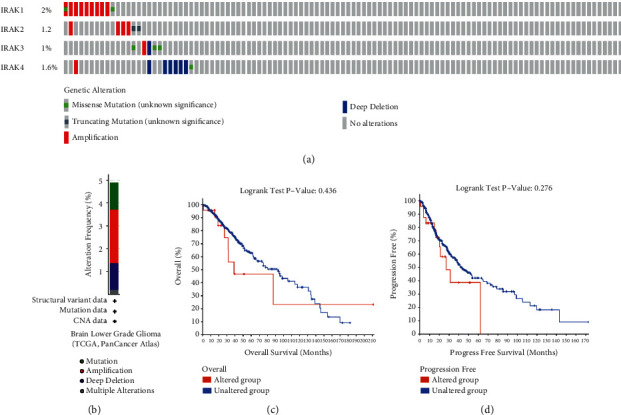
Genetic mutations and their association with LGG prognosis of IRAK family genes. (a, b) The mutation proportions and types of IRAK genes for LGG patients (TCGA, PanCancer Atlas) from OncoPrint of cBioPortal. (c) Kaplan–Meier survival curves of IRAK gene alterations and overall survival, as well as progression-free survival (d) of LGG patients.

To further explore the roles of the IRAK family comprehensively in LGG, we analyzed genetic alteration in the 4 genes and their correlations with OS and progression-free survival (PFS) using the cBioPortal online tool for LGG (TCGA, PanCancer Atlas), in which IRAK genes were altered in 25 samples out of 511 patients (5%). IRAK1 showed the highest mutation rate (2.5%), followed by IRAK4 (1.6%), IRAK2 (1.2%), and IRAK3 (1%) (Figure 9(a)). Our results uncovered amplifications, deep deletions, and mutations to be the dominating genetic mutation types for the IRAK family in LGG (Figure 9(b)). Further Kaplan–Meier plot and log-rank test exhibited the trend that patients with IRAKs alteration were related to a relatively poor prognosis, including OS (Figure 9(c)) and PFS (Figure 9(d)), although not statistically significant. These findings suggested that more emphasis is supposed to be put on exploring the genetic alterations of IRAK family genes in LGG.

### 3.6. IRAK1 Knockdown Inhibits Low-Grade Glioma Development In Vitro and In Vivo

**Figure 10:**
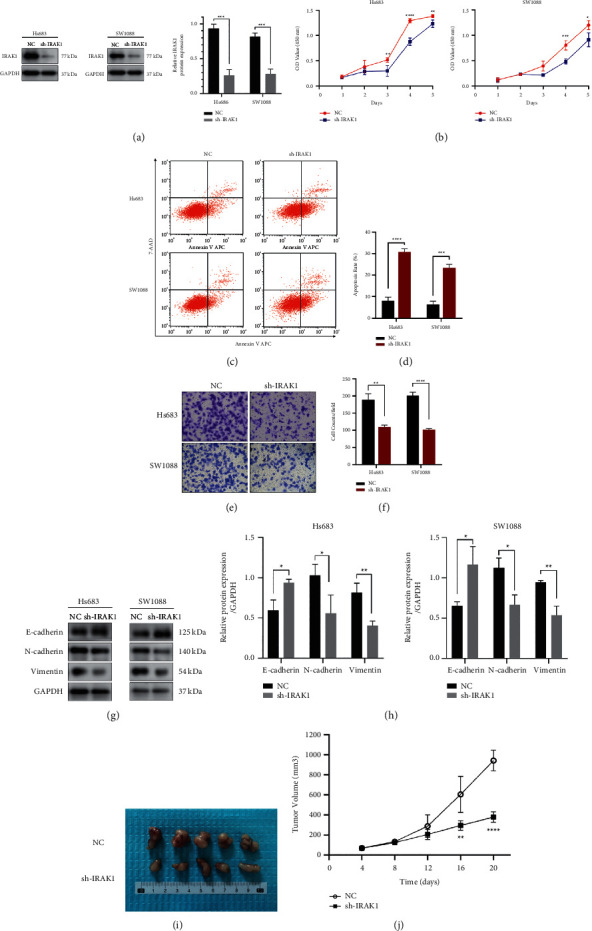
The effects of IRAK1 knockdown in vitro and in vivo assays in LGG. (a) Western blot analysis to validate the knockdown of IRAK1 in Hs683 and SW1088 glioma cells. (b) Cell proliferation of NC and sh-IRAK1 glioma cells was evaluated by CCK-8 assays (*n* = 5). In Hs683 cells, the OD values of NC vs sh-IRAK1 group were 0.516 ± 0.045 vs 0.299 ± 0.106 (day 3), 1.292 ± 0.042 vs 0.876 ± 0.074 (day 4), and 1.382 ± 0.031 vs 1.232 ± 0.072 (day 5). The OD values of SW1088-NC and SW1088-sh-IRAK1 cells were 0.393 ± 0.096 vs 0.216 ± 0.016 (day 3), 0.803 ± 0.093 vs 0.484 ± 0.048 (day 4), and 1.198 ± 0.090 vs 0.910 ± 0.138 (day 5). (c, d) The apoptosis rate of NC and sh-IRAK1 glioma cells was examined by flow cytometry (*n* = 3). (e) The migration ability of Hs683 and SW1088 cells with IRAK1 knockdown was tested by transwell assays (*n* = 3). The counts of migrated cells per field in Hs683-NC, Hs683-sh-IRAK1, SW1088-NC, and SW1088-sh-IRAK1 cells were 188.7 ± 17.6, 109.0 ± 6.2, 200.7 ± 10.3, and 101.3 ± 4.0, respectively. (f) The corresponding data are shown in a histogram. (g) Western blot assays of EMT-associated protein in NC and sh-IRAK1 glioma cells. (h) Relative protein abundance of EMT-related indicators was calculated by ImageJ. (i) Images of xenografts 20 days after inoculation of Hs683 cells transfected with sh-IRAK1 or the control vector. (j) The tumor volume was monitored every 4 days for 20 days (*n* = 5/group). The data are presented as mean ± SD.  ^*∗*^*P* < 0.05,  ^*∗∗*^*P* < 0.01,  ^*∗∗∗*^*P* < 0.001, and  ^*∗∗∗∗*^*P* < 0.0001.

Given that IRAK family genes were significantly higher expressed in LGG and closely associated with patient poor prognosis, especially for IRAK1, we suspected that IRAK1 might serve as an oncogene in LGG. To investigate whether IRAK1 can facilitate malignancy of LGG in vitro, tumor cell growth, apoptosis, and migration assays were conducted. Firstly, Hs683 and SW1088 glioma cell lines were transfected with IRAK1 knockdown lentivirus, which was verified using Western blot analysis (Figure 10(a) and Supplementary Figure S1). The results of CCK-8 assays showed that the proliferation rate was significantly diminished in sh-IRAK1 glioma cells compared to the control cells (Figure 10(b)). Additionally, apoptosis analyses showed that the percentage of cell apoptosis in Hs683-NC, Hs683-sh-IRAK1, SW1088-NC, and SW1088-sh-IRAK1 cells was 8.07 ± 1.72, 30.65 ± 1.51, 6.30 ± 1.66, and 23.38 ± 1.62, respectively (Figures 10(c) and 10(d)). These results suggested that IRAK1 silencing could induce glioma cells' apoptosis. To determine the effect of IRAK1 on glioma cell motility, cell migration was examined after the knockdown of IRAK1 in LGG cells by performing transwell assays. The results demonstrated that the migration ability of IRAK1 knockdown cells was significantly decreased (Figures 10(e) and 10(f)). Since EMT is a crucial mechanism involved in cancer cell invasion, migration, and metastasis, herein, we evaluated the expression of EMT-related proteins after knockdown of IRAK1 in LGG cell lines. Consistent with the data in transwell assays, IRAK1 silencing increased the expression of the epithelial marker E-cadherin and decreased the expression of the mesenchymal markers N-cadherin and Vimentin (Figures 10(g) and 10(h) and Supplementary Figure S2). To investigate the in vivo effect of IRAK1 silencing, IRAK1 knockdown Hs683 cells and negative control cells were subcutaneously injected into nude mice (*n* = 5 per group). We monitored the volume of xenograft tumors and found that the silencing of IRAK1 significantly slowed tumor growth in vivo (tumor volume in NC vs sh-IRAK1 group: 941.9 ± 104.4 vs 377.0 ± 52.5 mm^3^, Figures 10(i)and 10(j)). Taken together, these results suggested that IRAK1 inhibits apoptosis and augments malignancy of LGG in vitro and in vivo.

## 4. Discussion

Cancer has become a serious public health problem worldwide, leading to an escalating death rate of 9.6 million cases in 2018 [19]. Although various therapeutic methods have been adopted, the prognostic outcomes in multiple types of tumors are still unsatisfactory. Urgent requirements are raised to confirm key tumor-related genes to better understand cancer initiation, maintenance, and progression [20]. It is now widely accepted that inflammation contributes to cancer pathogenesis, to a certain extent. Not surprisingly, as an activator of IRAK signaling, the proinflammatory cytokine IL-1*β* participates in tumor growth, invasion, metastasis, angiogenesis, and chemoresistance [21, 22]. Similar roles have been observed in TLRs to promote tumor responses [23]. Thus, IRAK family kinases, essential mediators of IL1R and TLR-inflammatory signaling, may become potential chemotherapy targets. Though few previous studies have demonstrated the function of IRAK genes in specific tumors, system-level analyses are still lacking. Herein, by integrating data across multiple cancer types, we comprehensively explored the landscape of IRAK family genes in pan-cancer.

The aberrant expression levels of IRAK family members have been reported in some human cancers, such as colorectal cancer [24], melanoma [25], prostate cancer [26], and hepatocellular carcinoma [7]. First, we presented the transcription landscape of IRAK family genes across 33 cancer types, where no intrinsic unified pattern showed. For example, IRAK1 and IRAK4 were upregulated in most types of cancer compared with their corresponding normal tissues. Nevertheless, IRAK2 and IRAK3 display complex expression spectrum, suggesting the need for further study for each IRAK family gene as an entity. It was widely believed that TME acts as a vital role in tumorigenesis and progression [27, 28]. Based on mRNA expression profile, ESTIMATE algorithm could generate three scores to assess the abundance of immune infiltration, including Immune score (represents the immune cell in tumor niche), Stromal score (represents stromal presence in tumor tissue), and Estimate score (infers tumor purity) [29]. Our results presented the TME infiltration of IRAK genes in pan-cancer, where most of them showed positive correlations with Immune score, Stromal score, and Estimate score. Besides, IRAK genes tend to be higher expressed in the C6 immune type, which is dominated by TGF-*β* signature and showed more infiltrate distribution of type I and II T cells and a survival disadvantage [18]. These results hinted that IRAK genes can be potential targets to reshape TME and protect the tumors from progression and metastasis. Further, pan-cancer prognosis analysis indicated that IRAK genes were extensively involved in patient OS to varying degrees. Notably, the significantly predictive roles of higher expression levels of all IRAK family genes for worse patient survival in LGG attracted our attention immensely. Hence, more investigations of IRAK function and underlying mechanisms in LGG were performed subsequently.

Glioma, the most frequent primary malignancy in the central nervous system (CNS), can be classified into low grade (grades II and III) and high grade (grade IV, glioblastoma) by the World Health Organization (WHO) [30]. Low-grade glioma (LGG) is characterized as less aggressive compared with glioblastoma, accounting for 10–20% of all infiltrating primary brain tumors [31]. Patients with LGG have a median survival time of more than 7 years [32], who cannot be cured completely after surgery combined with standard chemoradiotherapy, due to substantial heterogeneity, therapeutic resistance, tumor recurrence, and progression [33]. Therefore, novel biomarkers are urgently needed to facilitate early diagnosis and predict the prognosis for patients with LGG [34]. Up to now, an increasing number of molecules, such as IDH-1 and 1p19q, have been discovered to play vital roles in LGG development [30, 35]. In this manuscript, we explored the function of IRAK family genes, especially for the oncogenic gene IRAK1 in LGG using bioinformatics analysis, in vitro experiment, and in vivo experiment.

In this study, we found that IRAK genes were higher expressed in LGG patients with WHO grade III than grade II from the TCGA database. IRAK1 and IRAK4 were significantly higher expressed in LGG samples than non-tumor tissues from the Rembrandt database. Additionally, IRAK genes were mainly positively correlated with immune infiltration. However, IRAK family genes had a significantly varying correlation with DNAss in different cancer types. The promoting or suppressing roles of IRAK family members in multiple tumors were controversial in the view of current research whereas there was little research investigating the function of IRAK family genes in glioma. Only a previous study identified that IRAK4 leads to chemoresistance to temozolomide in glioma cells [36]. Herein, we for the first time reported that IRAK1 was a novel oncogene in LGG, capable of inhibiting cell apoptosis and promoting glioma malignancy in vitro and in vivo. Epithelial-mesenchymal transition (EMT) is a crucial cascade process involved in the properties of tumor cell invasion and motility, wherein epithelial cells lose polarization and cell-cell tight junctions [37]. During EMT, tumor cells acquired highly aggressive capabilities and lost expression of epithelial markers, such as E-cadherin, and increased expression levels of mesenchymal markers, such as N-cadherin, Snail, and Vimentin [38]. We found that IRAK1 silencing attenuates cell migration and the EMT process in LGG.

In summary, our study revealed the expression profile of IRAK family genes with significant prognosis value in pan-cancer, especially in LGG. IRAK family genes were correlated with TME, Stemness score, and immune subtype. Moreover, the integrative analysis identified IRAK1 as a novel oncogene in LGG, which was subsequently verified in vitro and in vivo. These findings may provide insights for further investigations of the IRAK family genes as potential targets in pan-cancer.

## Figures and Tables

**Table 1:** The chromosomal locations of IRAK family members.

IRAK family proteins	IRAK1	IRAK2	IRAK3	IRAK4
Chromosomal locations	Xq28	3p25.3	12q14.3	12q12

**Table 2:** IRAK family genes were related to the prognosis risk in pan-cancer by conducting Cox analysis.

Gene	Cancer	HR	HR.95L	HR.95H	*P* value
IRAK1	ACC	1.714	1.113	2.64	0.014
	KICH	4.354	1.14	16.632	0.031
	KIRC	1.356	1.023	1.798	0.034
	LAML	2.478	1.609	3.814	3.78E-05
	LGG	2.036	1.425	2.908	9.26E-05
	LIHC	1.485	1.209	1.824	1.64E-04
	STAD	0.768	0.609	0.968	0.025
	THCA	2.333	1.073	5.076	0.033
	THYM	2.686	1.02	7.075	0.046
	UCEC	1.361	1.036	1.789	0.027
	UVM	3.202	1.44	7.122	0.004

IRAK2	BLCA	0.814	0.711	0.933	0.003
	LGG	1.9	1.502	2.403	8.87E-08
	LIHC	1.298	1.047	1.61	0.017
	PAAD	1.614	1.276	2.04	6.42E-05
	SKCM	0.654	0.531	0.805	6.24E-05
	TGCT	19.515	1.752	217.344	0.016
	THYM	3.52	1.276	9.711	0.015

IRAK3	BLCA	1.316	1.006	1.722	0.045
	HNSC	0.787	0.628	0.985	0.037
	KIRC	0.707	0.531	0.940	0.017
	LGG	1.948	1.338	2.836	5.03E-04
	PAAD	1.366	1.001	1.864	0.049
	THYM	2.912	1.167	7.263	0.022

IRAK4	BLCA	0.687	0.498	0.948	0.022
	KICH	4.675	1.076	20.322	0.040
	LGG	4.496	2.937	6.882	4.50E-12
	PAAD	0.541	0.311	0.940	0.029
	SKCM	0.706	0.558	0.892	0.004
	THYM	0.162	0.030	0.884	0.036
	UCEC	0.480	0.315	0.731	6.29E-04

## Data Availability

Data and materials are available upon reasonable request if applicable.

## Supplementary Materials

Supplementary Materials Supplementary Figure 1: scatter plots for Spearman correlation tests between IRAK gene expression and DNAss, Immune score, Stromal score, and Estimate score in three histology-based subtypes of LGG, including astrocytoma (A), oligodendroglioma (B), and mixed glioma (C). Supplementary Figure 2: differential IRAK family gene expression across immune infiltrate subtypes in three histology-based subgroups of LGG, including astrocytoma (A), oligodendroglioma (B), and mixed glioma (C). C3, inflammatory; C4, lymphocyte depleted; C5, immunologically quiet; C6, TGF-*β* dominant.  ^*∗*^*P* < 0.05,  ^*∗∗*^*P* < 0.01, and  ^*∗∗∗∗*^*P* < 0.0001. Supplementary Figure S1: full-size blots of Figure 10A. Supplementary Figure S2: full-size blots of Figure 10G.
