# Supplementary material for: Comprehensive Pan-Cancer Analysis of IRAK Family Genes Identifies IRAK1 as a Novel Oncogene in Low-Grade Glioma
Source: J Oncol. 2022 Feb 15;2022:6497241. doi: 10.1155/2022/6497241 (PMC8863493; doi:10.1155/2022/6497241)

Supplementary Fig. 1

Scatter plots for Spearman correlation tests between IRAK gene expression and DNAss, Immune score, Stromal score, and Estimate score in three histologic-based subtypes of LGG, including Astrocytoma (A), Oligodendroglioma (B), and mixed glioma (C).

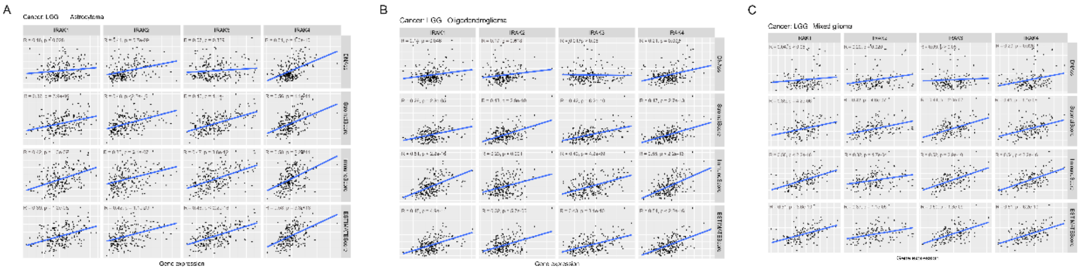

Supplementary Fig. 2

Differential IRAK family gene expression across immune infiltrate subtypes in three histologic-based subgroups of LGG, including Astrocytoma (A), Oligodendroglioma (B), and mixed glioma (C). C3, inflammatory; C4, lymphocyte depleted; C5, immunologically quiet; C6, TGF-β dominant. * P < 0.05, ** P < 0.01, **** P < 0.0001.

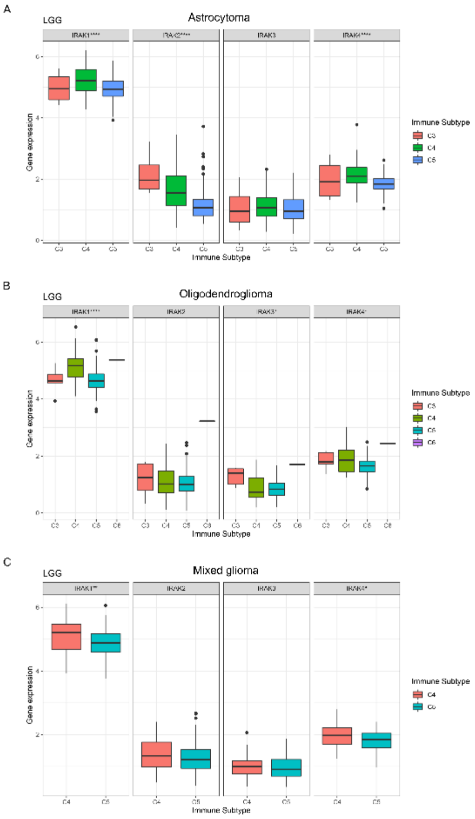

Supplementary Fig. S1 Full-size blots of Fig. 10A.

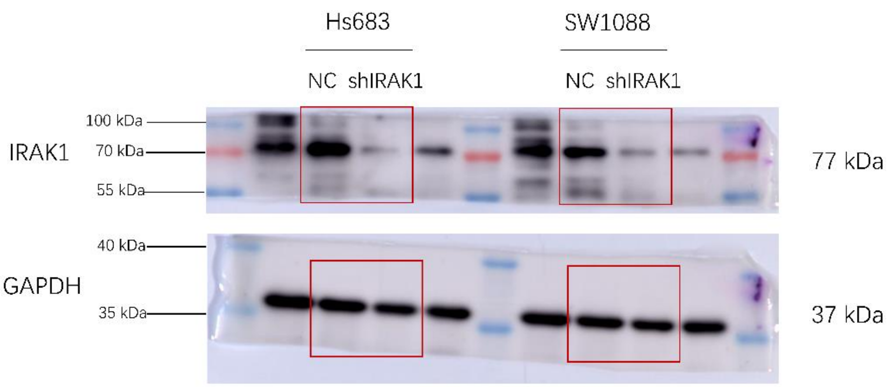

Supplementary Fig. S2 Full-size blots of Fig. 10G.

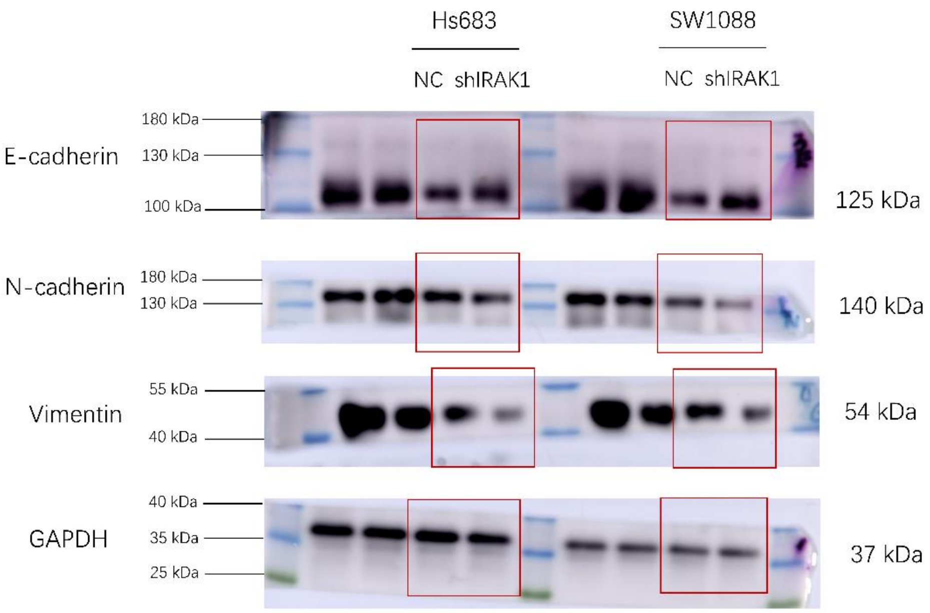

Supplement: Supplementary Materials — Supplementary Figure 1: scatter plots for Spearman correlation tests between IRAK gene expression and DNAss, Immune score, Stromal score, and Estimate score in three histology-based subtypes of LGG, including astrocytoma (A), oligodendroglioma (B), and mixed glioma (C). Supplementary Figure 2: differential IRAK family gene expression across immune infiltrate subtypes in three histology-based subgroups of LGG, including astrocytoma (A), oligodendroglioma (B), and mixed glioma (C). C3, inflammatory; C4, lymphocyte depleted; C5, immunologically quiet; C6, TGF-β dominant. ∗P < 0.05, ∗∗P < 0.01, and ∗∗∗∗P < 0.0001. Supplementary Figure S1: full-size blots of Figure 10A. Supplementary Figure S2: full-size blots of Figure 10G. [file 6497241.f1.pdf]
